# Impact of atrial functional substrate in patients with atrial fibrillation: The potential utility of decremental evoked potential mapping in the atrium

**DOI:** 10.1016/j.hroo.2024.11.015

**Published:** 2024-11-22

**Authors:** Yasuhito Kotake, Fumiyasu Hirano, Shunsuke Kawatani, Aiko Takami, Takuya Tomomori, Akihiro Okamura, Masaru Kato, Kazuhiro Yamamoto

**Affiliations:** 1 Department of Cardiovascular Medicine, Endocrinology and Metabolism, Faculty of Medicine, Tottori University, Yonago, Japan; 2 Department of Cardiology, Tottori Prefectural Central Hospital, Tottori, Japan

**Keywords:** Atrial fibrillation, Functional substrate, Decremental evoked potential, Pericardial fat volume, Electrophysiological remodeling

## Abstract

**Background:**

Decremental evoked potential (DEEP) is one of the functional substrates mainly used in the field of ventricular arrhythmias, which is suggested to be a critical target of reentrant ventricular tachycardia.

**Objective:**

The purpose of this study is to investigate the characteristics of patients with atrial functional substrates expressed by DEEP and their clinical significance.

**Methods:**

Patients presenting for atrial fibrillation (AF) ablation from April 2023 to March 2024 at Tottori University Hospital were analyzed. After cryoballoon pulmonary vein isolation, DEEP was evaluated at the left atrial roof and posterior wall by extrastimulus pacing maneuvers. To verify the clinical significance of atrial DEEP, the relationship between atrial DEEP and various clinical valuables including the pericardial fat volume and clinical outcomes was assessed.

**Results:**

A total of 102 patients were included and 45% had persistent AF. Fifty-three percent of patients exhibited DEEP properties. DEEP was more prevalent in patients with persistent AF (61% vs 39%, *P <* .001), higher brain natriuretic peptide levels (194 [interquartile range (IQR) 106–270] pg/mL vs 90 [IQR 23–174] pg/mL, *P =* .01), and a greater pericardiac fat volume (112 [IQR 63–76] cm^3^ vs 75 [IQR 53–95] cm^3^, *P =* .001). The patients with atrial DEEP had more early AF recurrence after ablation procedure (*P <* .001).

**Conclusion:**

This study demonstrated a correlation between atrial DEEP and longer duration of AF, higher brain natriuretic peptide levels, greater pericardial fat volume, and more early AF recurrence, suggesting that DEEP reflects a certain aspect of atrial electrophysiological remodeling and is a potential ablation target for AF.

## Graphical abstract

**Figure:**
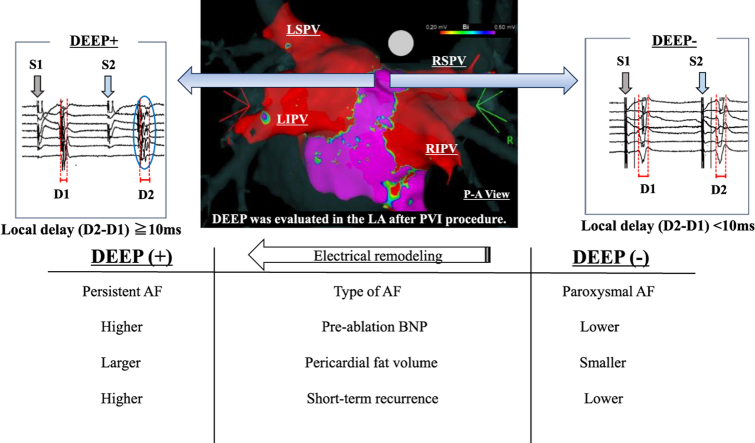

**Key Findings**

▪ Atrial decremental evoked potential (DEEP) was more prevalent in patients with persistent atrial fibrillation (AF), higher preablation brain natriuretic peptide level and greater amount of pericardial fat.
▪ During follow-up period, patients with atrial DEEP had a significantly higher early AF recurrence after pulmonary vein isolation.
▪ DEEP might reflect a certain aspect of atrial electrophysiological remodeling and potential ablation target for AF.

## Introduction

Cryoballoon pulmonary vein isolation (PVI) has emerged as a cornerstone strategy for the management of atrial fibrillation (AF). While initial techniques sought to target pulmonary vein (PV) as an arrhythmogenic substrate, contemporary studies have focused on identification and elimination of arrhythmogenic substrate beyond PV.^1^^,^^2^ That is because arrhythmogenic substrate progressively extends from the PV itself to the entire left atrium (LA), including right atrium and superior vena cava. In this context, characterization of arrhythmogenic substrate in patients with AF has emerged as a crucial determinant of ablation success. Recently, multipolar mapping catheters with small and closely spaced electrodes have revealed finer details of the underlying substrate. Further, arrhythmogenic substrates sometimes act as functional substrates which are invisible during stable sinus rhythm or a pacing rhythm.^3^ These functional substrates can be distinguished by using extrastimulus pacing maneuvers with their decremental conduction property. Decremental evoked potential (DEEP) is one of the functional substrates mainly used in the field of ventricular arrhythmias, which is suggested to be a critical target of reentrant ventricular tachycardia.^4^ However, limited studies have assessed the potential utility of DEEP mapping in the atrium. We hypothesize that atrial DEEP in patients with AF reflects a certain aspect of atrial electrophysiological remodeling and potential ablation targeting. The purpose of this study is to investigate the characteristics of patients with the atrial functional substrate expressed by DEEP and their clinical significance.

## Methods

### Study participants

Prospectively collected data on patients who underwent cryoballoon catheter ablation of AF at Tottori University Hospital between April 2023 and March 2024 were analyzed.

Written informed consent was obtained from all cases. This study was approved by the Human Research Ethics Committee of Tottori University Hospital and complies with the Declaration of Helsinki.

Exclusion criteria were as follows: (1) patients <18 years of age; (2) patients who had experienced previous LA ablation or LA surgery; (3) patients with substrate-based structural heart disease (left ventricular ejection fraction [LVEF] <50%); (4) patients with severe degenerative mitral regurgitation or stenosis; and (5) AF due to reversible causes (eg, hyperthyroidism). All patients underwent echocardiography and cardiac computed tomography (CT) to screen for the presence of structural heart disease as a part of routine care.

### Mapping protocol

#### Catheter ablation strategy and mapping strategy

Our ablation strategy has previously been described.^5^ In brief, patients were sedated with conscious sedation. Catheters were introduced into the LA transseptally. Systemic anticoagulation was administered after sheath insertion using intravenous unfractionated heparin to maintain an activated clotting time >300 ms prior to LA access. Antiarrhythmic drug therapy was withheld for 5 half-lives preablation. An over-the-wire 15-F steerable sheath (FlexCath or FlexCath Advance Steerable Sheath; Medtronic) was used to introduce the 28-mm cryoballoon ablation catheter (Arctic Front Advance; Medtronic) into the LA. A spiral mapping catheter (Achieve; Medtronic) was used to advance the cryoballoon and to map the target PV potentials. Before each ablation, the cryoballoon catheter was inflated and advanced toward the antral surface of the PV. The cryoapplication was administered after confirmation of an antral occlusion of the PV. To avoid phrenic nerve injury, diaphragmatic compound motor action potentials were monitored with phrenic nerve pacing during each cryoballoon application. The procedural endpoints were defined as the establishment of bidirectional PV-LA block, which was verified via an Octaray multielectrode mapping catheter (Biosense Webster). If electrical isolation was not achieved after cryoballoon applications (180 seconds for each application) at each vein, additional touch-up ablation was performed using conventional radiofrequency. In patients with persistent AF, the LA roof line was also created using the cryoballoon. After PVI, then anchoring the Achieve mapping catheter in the right superior PV, sequential retraction and posterior rotation of the balloon were employed to create overlapping and contiguous stamp lesions spanning the LA roof to create the roof line.

Three-dimensional electroanatomic mapping of the LA was depicted with a multipolar mapping catheter (Octaray; 2-2-2 mm electrode spacing) using the CARTO electroanatomic mapping system (version 7; Biosense Webster). Points were acquired evenly across the entire LA. Strict criteria were employed to account for the lack of tissue contact data on the multipolar mapping catheter. Tissue contact was also evaluated by Tissue Proximity Indication (Biosense Webster), which reflects real-time assessment of diagnostic catheter electrodes’ proximity to cardiac tissue based on impedance measurements. Point collection was performed only by experienced operators after careful assessment of tactile catheter pressure, fluoroscopic motion, and application of an internal point filter to within 10 mm of the chamber surface geometry and ensuring tissue contact using intracardiac echocardiography. The mitral annulus was excluded from analysis. Bipolar voltage was defined as the peak to peak electrogram voltage. The low-voltage area was defined as bipolar voltage <0.5 mV.

#### Programmed electrical stimulation protocol and definition of DEEP

**Figure 1:**
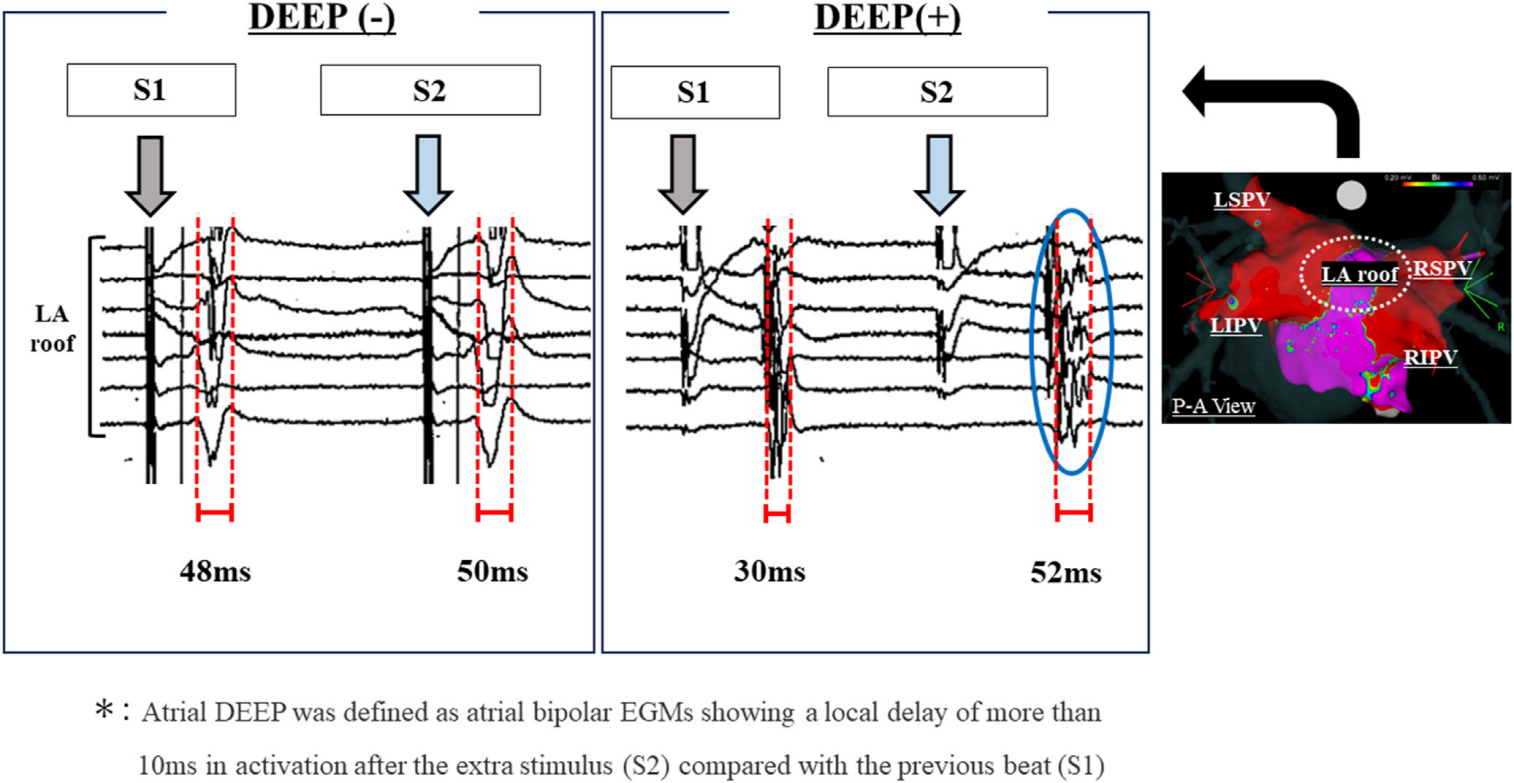
Definition of atrial decremental evoked potential (DEEP). Atrial DEEP was defined as atrial bipolar electrograms (EGMs) exhibiting a local activation delay of more than 10 ms after an extrastimulus (S2) as compared with the previous beat (S1). (Left) Several EGMs recorded during S1 of 600 ms and after S2 (10 ms above the atrial refractoriness) exhibiting a minimal change (<10 ms) in EGM duration, indicating DEEP–. (Right) In contrast, several EGMs showed a notable decrement of more than 10 ms after S2 as compared with the previous beat (S1) defined as DEEP+. These were recorded on the left atrial roof. LA = left atrial; LIPV = left inferior pulmonary vein; LSPV = left superior pulmonary vein; RIPV = right inferior pulmonary vein; RSPV = right superior pulmonary vein.

DEEP was evaluated at the LA roof and posterior wall from 2 different pacing sites (endocardially from the LA appendage [LAA], epicardially from the proximal coronary sinus [CS]) after PVI procedure. In patients with persistent AF, DEEP mapping was performed before creating LA roof line. Extrastimulus electrograms (EGMs) were individually measured and analyzed. If the patient remained in AF after PVI, synchronized direct current cardioversion was performed to restore sinus rhythm. The EGMs during S1 600-ms drive train of 4 beats and after an extrastimulus (S2 at +10 ms above atrial refractoriness) were studied at each location and assessed for DEEP properties. Based on the previous study, atrial DEEP was defined as atrial bipolar EGMs showing a local delay of more than 10 ms in activation after the extra stimulus compared with the previous beat (Figure 1).^6^ DEEP sampling was obtained at the 2 different sites including LA roof and posterior wall. In this study, patients in whom DEEP was confirmed in at least 1 of the LA roof or posterior regions by CS or LAA pacing were defined as DEEP positive (DEEP +). Patients in whom DEEP was not confirmed in the LA roof and posterior regions by both CS and LAA pacing were defined as DEEP negative (DEEP–).

#### Pericardial fat volume

Pericardial fat volumes were measured offline by commercially available software package zioM900 (Zio Software) using an algorithm of attenuation on cardiac CT as previously described.^7^^,^^8^ Using contrast-enhanced CT, an axial view of the heart was created with 0.6-mm slice thickness. Tissue between –200 and –50 Hounsfield units was defined as fat tissue and measured semi-automatically. Regions of interest containing pericardial fat were created from the inferior of pulmonary artery to the left ventricular (LV) apex.

#### Follow-up

The hospital medical records and outpatient clinic assessments were used to complete the clinical follow-up. Patients were followed by symptoms, electrocardiography at 1, 3, and 6 months after discharge. Furthermore, AF recurrences were also assessed by implantable loop recorders or pacemakers/defibrillators, when available. Any documented AF lasting >30 seconds detected using 12-lead electrocardiography or other appropriate tests was considered to be a recurrence.

### Statistical analysis

All statistical analyses were performed with JMP version 14 software (SAS Institute). The normality distribution of continuous data was tested using Shapiro–Wilk test. Continuous variables were expressed as the mean ± SD if normally distributed, and median and interquartile range (IQR) or full ranges were used if the data were clearly skewed. Continuous variables were compared using the Student *t* test when normally distributed or a Mann-Whitney *U* test when they were not normally distributed. A chi-square test was used when comparing categorical variables or a Fisher's exact test when required. A 2-tailed *P* value <.05 was considered statistically significant. McNemar’s test was used for the regional distribution analysis of DEEP property.

## Results

### Study participants

**Figure 2:**
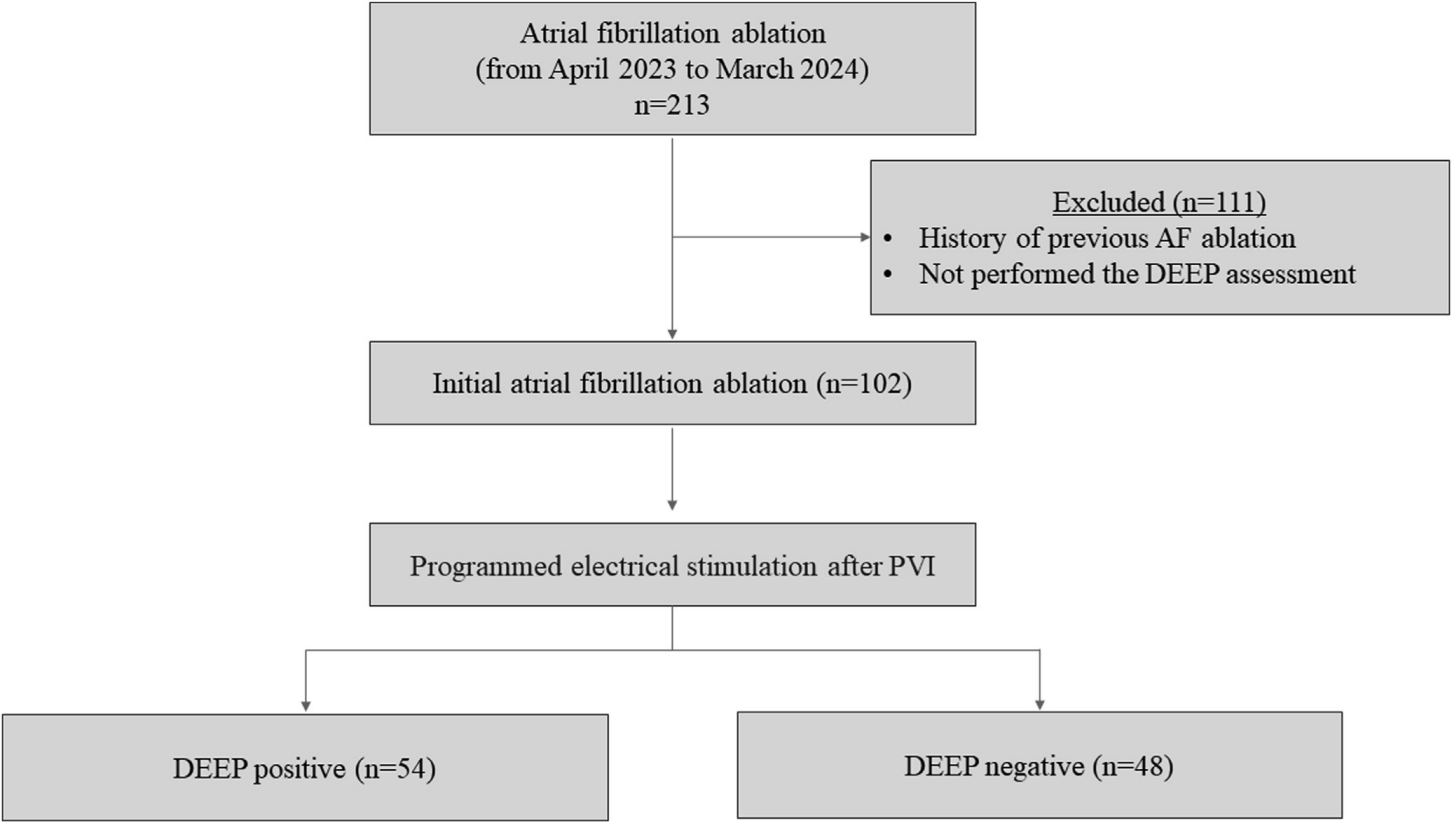
Study enrollment. During study periods, 213 patients who underwent atrial fibrillation (AF) ablation at Tottori University Hospital were reviewed. A total of 111 patients were excluded due to the exclusion criteria. As a result, 102 patients were included in this study. Fifty-four patients exhibited decremental evoked potential (DEEP) properties, while 48 patients did not exhibit DEEP properties.

Of the 213 consecutive patients who underwent AF ablation during the study period, 102 patients fulfilled the inclusion criteria (Figure 2). Baseline characteristics are shown in Table 1. The median age of the population was 69 years, and there were 74 (73%) males with a mean LVEF of 59%. Forty-five percent had persistent AF.

**Table 1:** Baseline characteristics (N = 102)

Age, y	69 (63–75)
Male	74 (73)
Body mass index, kg/m^2^	24 (22–27)
Hypertension	61 (60)
Diabetes	21 (21)
Dyslipidemia	39 (38)
Smoking	24 (24)
Persistent AF	46 (45)
BNP, pg/mL	136 (34–228)
Ccr, mL/min	71 (57–91)
Echocardiography	
LV ejection fraction, %	59 (53–63)
Left atrial dimension, mm	40 ± 6
Cardiac computed tomography	
Pericardial fat volume, cm^3^	94 (66–133)
Electrophysiologic study	
Atrial DEEP+	54 (53)

Values are median (interquartile range), n (%), or mean ± SD.

AF = atrial fibrillation; BNP = brain natriuretic peptide; Ccr = creatinine clearance; DEEP = decremental evoked potential; LV = left ventricular.

### Analysis of DEEP mapping

Fifty-three percent of patients exhibited DEEP properties at the LA roof and/or posterior wall. With conventional bipolar voltage setting (low-voltage area was defined as bipolar voltage < 0.5 mV), there was hardly any obvious low-voltage area within the LA in our study. In particular, in the areas where DEEP mapping was performed (ie, the LA roof and posterior wall), low LA voltage was not identified in any patient in this study. Table 2 shows the baseline characteristics comparing patients in the DEEP+ and DEEP– groups. Atrial DEEP was more prevalent in patients with persistent AF (persistent AF 61% vs paroxysmal AF 27%, *P =* .001). In terms of the brain natriuretic peptide (BNP) level, patients with DEEP had higher BNP levels (194 [IQR 106–270] pg/mL vs 90 [IQR 23–174] pg/mL, *P =* .01). Pericardiac fat volume was also assessed using cardiac CT in this study. Patients in the DEEP+ group had a significantly greater pericardial fat volume than those in the DEEP– group (112 [IQR 85–148] cm^3^ vs 75 [IQR 53–95] cm^3^, *P =* .001).

**Table 2:** Baseline characteristics comparing patients with DEEP+ and DEEP–

	DEEP+ (n = 54)	DEEP– (n = 48)	*P* value
Age, y	71 (63–76)	68 (60–74)	.27
Male	39 (72)	35 (73)	.94
Body mass index, kg/m^2^	23 (21–25)	25 (23–27)	.09
Hypertension	36 (67)	25 (52)	.13
Diabetes	9 (17)	12 (25)	.30
Dyslipidemia	15 (28)	22 (46)	.06
Smoking	12 (22)	12 (25)	.74
Persistent AF	33 (61)	13 (27)	.001∗
BNP, pg/mL	194 (106–270)	90 (23–174)	.01∗
Ccr, mL/min	69 (56–90)	73 (59–91)	.19
Echocardiography			
LV ejection fraction, %	60 (54–63)	58 (51–62)	.16
Left atrial dimension, mm	40 ± 7	39 ± 4	.11
Cardiac computed tomography			
Pericardial fat volume, cm^3^	112 (85–148)	75 (53–95)	.001∗

Values are median (interquartile range), n (%), or mean ± SD.

AF = atrial fibrillation; BNP = brain natriuretic peptide; Ccr = creatinine clearance; DEEP = decremental evoked potential; LV = left ventricular.

∗ *P* values were consiered statistically significant when .05 or less.

### Regional distribution of DEEP determined by endocardial and epicardial pacing

**Figure 3:**
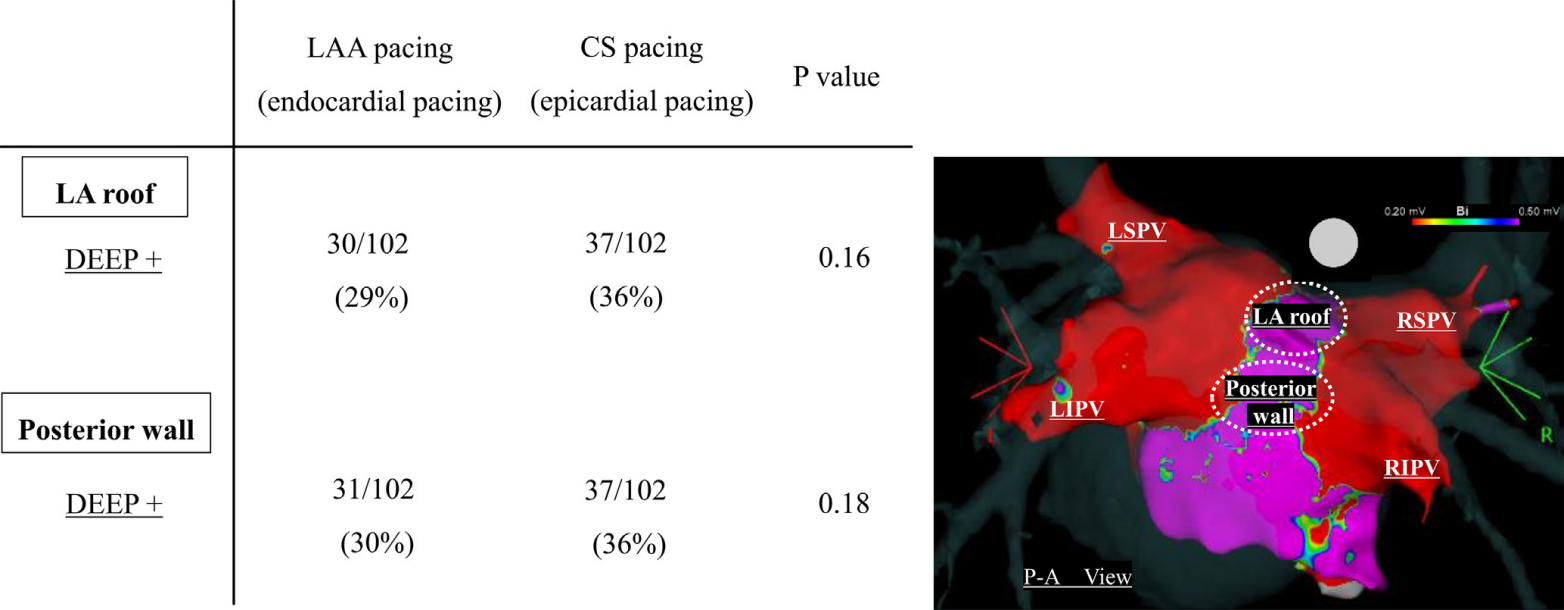
Regional distribution of decremental evoked potential (DEEP) according to the 2 different pacing sites. DEEP mapping was performed at the left atrial (LA) roof and posterior wall from 2 different pacing sites (endocardially from the left atrial appendage [LAA] and epicardially from the proximal coronary sinus [CS]). DEEP was equally distributed between the LA roof and posterior wall. Per analysis according to the pacing site, there was a tendency for DEEP to be observed more frequently with CS pacing than LAA pacing; however, there was no significant difference. LIPV = left inferior pulmonary vein; LSPV = left superior pulmonary vein; RIPV = right inferior pulmonary vein; RSPV = right superior pulmonary vein.

DEEP mapping was performed at the LA roof and posterior wall from 2 different pacing locations (endocardially from the LAA and epicardially from the proximal CS) in this study. The regional distribution of DEEP according to the 2 different pacing sites is shown in Figure 3. When analyzed by the anatomical location, DEEP were equally distributed between LA roof and posterior wall. When analyzed by the pacing site, there was a tendency for DEEP to be observed more frequently with CS pacing than LAA pacing; however, there was no significant difference.

### Clinical outcome

The patients with atrial DEEP had more early recurrence of AF within 3 months after the ablation procedure than those without atrial DEEP (50% vs 10%, *P <* .001). Regarding recurrence of AF between 3 and 6 months, there was no significant difference between groups (15% vs 6%, *P =* .21). Those results were consistent even in separate analysis of patients with paroxysmal AF (within 3 months: 38% vs 6%, *P =* .004; from 3 to 6 months: 5% vs 3%, *P =* .86) and persistent AF (within 3 months: 58% vs 23%, *P =* .04; from 3 to 6 months: 21% vs 15%, *P =* .80) (Table 3).

**Table 3:** Clinical outcome depending on the presence of DEEP

AF recurrence	DEEP+	DEEP–	*P* value
Among all 102 patients	n = 54	n = 48	
Within 3 mo	27 (50)	5 (10)	.001∗
From 3 to 6 mo	8 (15)	3 (6)	.21
Among 56 patients with paroxysmal AF (ablation strategy: PVI only)	n = 21	n = 35	
Within 3 mo	8 (38)	2 (6)	.004∗
From 3 to 6 mo	1 (5)	1 (3)	.86
Among 46 patients with persistent AF (ablation strategy: PVI + roof line)	n = 33	n = 13	
Within 3 mo	19 (58)	3 (23)	.04∗
From 3 to 6 mo	7 (21)	2 (15)	.80

Values are n (%).

AF = atrial fibrillation; DEEP = decremental evoked potential; PVI = pulmonary vein isolation.

∗ *P* values were consiered statistically significant when .05 or less.

## Discussion

In the field of ventricular arrhythmia, numerous studies have reported the usefulness of functional substrate mapping for the ventricular tachycardia ablation. Jackson and colleagues^4^ reported that the concept of DEEP, whereby local abnormal potentials with decremental conduction properties were found to have greater specificity for a critical site of the ventricular tachycardia circuit than late potentials. DEEP mapping assesses the decremental properties of myocardial potentials based on the fundamental understanding that areas of decremental conduction will cause unidirectional block and slow conduction, which are prerequisites for re-entrant arrhythmias. Porta-Sánchez and colleagues^9^ reported that DEEP focused ablation resulted in a reduction in ventricular tachycardia burden in patients with ischemic cardiomyopathy. However, limited data have reported the usefulness of functional substrate mapping for AF ablation. The present study validates the clinical significance of atrial functional substrate expressed by DEEP in patients with AF. The main findings of this study are as follows: (1) atrial DEEP was more prevalent in patients with persistent AF and higher preablation BNP level; (2) patients with atrial DEEP had significantly greater amount of pericardial fat compared with those without atrial DEEP; (3) there were no significant differences in DEEP identification according to the pacing site or anatomical location, but there was a tendency for DEEP to be observed more frequently with CS pacing (epicardial pacing) than LAA pacing (endocardial pacing); and (4) during the follow-up period, patients with atrial DEEP had a significantly higher early recurrence rate.

In this study, atrial DEEP was more common in patients with persistent AF and higher BNP level. That suggests that atrial DEEP increases with AF burden and reflects a certain aspect of atrial electrical remodeling. AF substrate progresses with the advancement of atrial structural and electrical remodeling, resulting in AF perpetuation and recurrence. In that context, atrial DEEP might be linked to AF pathology.^10^ Furthermore, there were no differences in cardiac function or morphological abnormalities, such as LVEF or LA dimension between patients with and without DEEP properties. Considering that DEEP was detected in the normal range of bipolar voltage (1.5 mV), those finding may support the idea that DEEP reflects potential and functional substrate prior to the development of structural abnormalities.

Second, patients with atrial DEEP had significantly greater amount of pericardial fat than those patients without atrial DEEP in our study. Pericardial fat is considered an electrical active organ, some of which is in direct contact with the myocardium, ganglionated plexi, and adipocytes as epicardial adipose tissue.^11^ Previous studies have reported that pericardial fat affects the myocardium itself such as causing electrical conduction disturbances or inflammation.^12^ Those previous studies and our data suggest that pericardial fat is directly related to LA DEEP properties.

Third, there was no significant difference in DEEP identification according to the pacing site or anatomical location; however, there was a tendency for DEEP identification to be higher by CS pacing (epicardial pacing) than LAA pacing (endocardial pacing). This is also concordant with a previous study that assessed atrial DEEP.^6^ This result may partly explain the correlation between the pericardial fat volume and DEEP identification. Considering that epicardial fat is involved in the development of atrial functional substrate, it is reasonable that DEEP is identified more frequently during epicardial pacing than endocardial pacing. Due to the small sample size in this study, no statistically significant differences were demonstrated. Further analysis with a larger number of subjects is needed.

Last, patients with atrial DEEP had significantly higher early AF recurrence rate than those without atrial DEEP after the AF ablation. In this study, patients with atrial DEEP tended to have a longer duration of AF, a greater amount of pericardial fat, and higher preablation BNP levels. Historical studies have shown that each of these factors is independently associated with AF recurrence after catheter ablation.^13^, ^14^, ^15^ Therefore, it is consistent to assume that DEEP+ patients have a higher incidence of AF recurrence after ablation. However, regarding long-term outcome over 3 months, there was no significant difference in recurrence rate between the groups. In the subanalysis according to the AF duration (paroxysmal AF and persistent AF), there was no statistical difference in the recurrence rate between paroxysmal AF and persistent AF depending on whether or not there was a DEEP identification; however, the difference in early recurrence rate was smaller in patients with persistent AF than paroxysmal AF. This is thought to be partly due to the fact that roof lines were created in patients with persistent AF in addition to PVI. Although AF recurrence was not completely suppressed, the difference in early recurrence rate seemed to be somewhat smaller after creating a roof line in patients with persistent AF. To verify the effectiveness of additional ablation procedures in patients with DEEP properties, longer term follow-up in larger cohort will be required.

So far, systematic data on atrial DEEP mapping after AF ablation are limited. The only study on atrial DEEP mapping was reported by Montañés and colleagues.^6^ They concluded that atrial DEEP mapping is useful to highlight areas with a tendency for unidirectional block and re-entry initiation. Our data are consistent with that previous study and provide further insight into the clinical significance of atrial DEEP by showing their relationship with the time course of AF and pericardial fat volume.

### Limitations

First, there might have been some hidden bias due to the small sample size. Second, pericardial fat volume was not measured directly adjunct to LA site, but rather at the area around the whole heart semi-automatically identified by a Hounsfield unit ratio between –200 and –50. Third, DEEP mapping was evaluated only at the LA roof and posterior wall in this study. This is because the LA roof and posterior wall are common areas where arrhythmogenic substrate is frequently observed, especially in patients with persistent AF. Further, a detailed mapping protocol (such as adding evaluation of the anterior wall, septum, and right atrium) would require a longer time, which was not suitable for clinical research in daily practice. Fourth, implantable electrocardiograms and/or long-term electrocardiogram monitoring were not available in all cases. This may have led to an underestimation of AF recurrence. Last, follow-up data are only for 6 months in this study. It is controversial to define AF recurrence after PVI ablation within 3 months. However, recent studies have reported that recurrence during this period is associated with long-term AF recurrence.^16^ To further validate the clinical significance of atrial DEEP, longer follow-up data and data on the intervention for atrial DEEP will be needed.

## Conclusion

This study demonstrates a correlation between atrial DEEP and longer AF duration, greater amount of pericardial fat, and more early recurrence of AF, suggesting that DEEP reflects a certain aspect of atrial electrophysiological remodeling and potential ablation target for AF. Further research will be needed to validate the clinical significance of atrial DEEP.
